# Correlation of acoustic voice analysis and Voice Handicap Index in patients with postoperative unilateral vocal cord paralysis after thyroid surgery

**DOI:** 10.1590/1414-431X2024e13528

**Published:** 2024-06-17

**Authors:** Yanrui Bian, Jingmiao Wang, Haizhong Zhang, Xiaoyan Yin, Yubo Zhang

**Affiliations:** 1 Department 1 of Otolaryngology, The Second Hospital of Hebei Medical University, Shijiazhuang, Hebei, China

**Keywords:** Thyroid surgery, Vocal cord paralysis, Voice Handicap Index, Acoustic voice analysis, Voice Handicap Index parameters

## Abstract

Unilateral vocal cord paralysis is frequently observed in patients who undergo thyroid surgery. This study explored the correlation between acoustic voice analysis (objective measure) and Voice Handicap Index (VHI, a self-assessment tool). One hundred and forty patients who had thyroid surgery with or without postoperative unilateral vocal cord paralysis (PVCP and NPVCP) were included. The patients were evaluated by the VHI and Dysphonia Severity Index (DSI) tools. VHI scores were significantly higher in PVCP patients than in NPVCP patients. Jitter (%) and shimmer (%) were significantly increased, whereas DSI was significantly decreased in PVCP patients. Receiver operating characteristics curve revealed that VHI scores were associated with the diagnosis of PVCP, of which VHI total score yielded an area under the curve (AUC) of 0.81. Among acoustic parameters, DSI was highly associated to PVCP (AUC=0.82, 95%CI=0.75 to 0.89). Moreover, we found a correlation between VHI scores and voice acoustic parameters. Among them, DSI had a moderate correlation with functional and VHI scores, as suggested by an R value of 0.41 and 0.49, respectively. VHI scores and acoustic parameters were associated with the diagnosis of PVCP.

## Introduction

Vocal cord paralysis is a disorder in which the vocal cord cannot move due to nerve damage (1,2). It is characterized by a number of symptoms including aspiration, dyspnea, dysphonia, hoarseness, and inability to speak (1-3). A series of conditions including surgery (thyroid surgery and mechanical ventilation, etc.), neurological conditions, infections, and tumors can cause vocal cord paralysis (3,4). Vocal cord paralysis can occur in both vocal folds (bilateral paralysis) or in a single vocal fold (unilateral paralysis), with unilateral paralysis being more common than bilateral paralysis (4,5). In addition, it has been reported that left vocal cord paralysis is more frequently observed than right vocal cord paralysis (5,6). Failure in diagnosing vocal cord paralysis in the initial stage may lead to more severe symptoms and cancerous tumors (3). Moreover, vocal cord paralysis is associated with an increased risk of other diseases including cardiovascular diseases and cancers, and affects the overall quality of life (7-9).

Currently, voice disorders can be assessed by a series of subjective and objective methodologies including laryngoscopy, auditory perception, acoustic analysis, as well as by questionnaire-based self-assessment (10,11). Objective assessments can provide better information about voice function and quality (11) than subjective measures. It is known that the patient's physical and mental stress can negatively affect the vocal cord (12). Therefore, subjective assessments are also important for the evaluation of vocal disorders by providing complementary information for objective assessments. For instance, the Voice Handicap Index (VHI) is commonly used as a self-assessment methodology for evaluating patients' perceptions of their vocal conditions (Supplementary Table S1) (13).

Voice disorders have been frequently observed in patients who have had thyroid surgery (14,15). Among them, vocal cord paralysis is one of the most commonly observed complications after thyroid surgery (16). Several previous prospective studies found that the incidence of vocal cord paralysis ranges from 3.5 to 6.6% in patients who have had thyroid surgery (17). Interestingly, bilateral vocal cord paralysis had an incidence of only 0.58% among those patients, suggesting that unilateral vocal cord paralysis is more commonly observed after thyroid surgery.

In this study, we aimed to explore the diagnostic values of subjective (VHI) and objective (acoustic analysis) voice assessments for postoperative unilateral vocal cord paralysis (PVCP) in patients after thyroid surgery. We also explored the correlation between VHI and acoustic voice parameters.

## Material and Methods

### Participants

All participants enrolled in this study had thyroid surgery at the Second Hospital of Hebei Medical University and read and signed the informed consent form. Participants included in this study met the following criteria: 1) vocal disorder reported by the participants within 1 year after thyroid surgery; 2) no cognitive impairment; and 3) ability to understand and complete the VHI. The patients had paralysis at the time of recording, and it took three days from diagnosis to recording. Neither voice improvement nor voice deterioration occurred before the recordings. The patients did not have any surgery or voice therapy before the recordings. After study inclusion, a dynamic laryngoscope was used to diagnose PVCP or NPVCP. Overall, seventy-eight patients were diagnosed with PVCP and sixty-two patients were diagnosed with NPVCP. VHI questionnaires were filled out by the patients on the first day of the recordings. The study was approved by the Second Hospital of Hebei Medical University.

Patients were excluded from this study if they had any of the following: 1) lesions of the vocal cords before surgery; 2) thyroid nodules on the tracheal ring or on the recurrent laryngeal nerves; 3) tracheostomy surgery; 4) vocal cord motion disorder before surgery; and 5) other neck diseases.

### VHI self-assessment

To evaluate the impact of the vocal cord disorder on the quality of life, the VHI questionnaire was completed by the participants. The VHI questionnaire contains functional, physical, and emotional modules, with 10 questions each (Supplementary Table S1). All questions were answered on a 5-point Likert scale to measure the attitude and opinion of the participants (0 represents never and 4 represents always). A higher score indicates greater impact of the vocal disorder.

### Acoustic assessment

Voice acoustic recordings were determined by acoustic analysis software (DIVAS2.5, Germany) in a room with a background noise level of less than 45 dB. The mouth-to-microphone distance was set to 30 cm. Subsequently, the participant was instructed to generate three vocal utterances with a consistent and steady tone. Samples were then acquired during the periods of stable vocalization. Acoustic parameters, encompassing jitter (%), shimmer (%), and the Dysphonia Severity Index, were subsequently obtained and calculated based on the recorded data.

### Data analysis

The data are shown in box plots with all the data points. Mann-Whitney test was performed to compare the difference between two groups of individuals. Receiver operating characteristics (ROC) curves were performed to evaluate the diagnostic values of each voice acoustic analysis parameter on PVCP. Spearman correlation analysis was used to determine the association between the DSI parameter and the VHI parameter. A P-value less than 0.05 was considered significant.

## Results

### Patient characteristics

Patient characteristics are summarized in Table 1. In the NPVCP group, fifty-five patients (88.7%) had papillary thyroid carcinoma and seven patients had nodular goiter (11.3%). In the PVCP group, seventy-two patients (92.3%) had papillary thyroid carcinoma and six patients had nodular goiter (7.7%). Thirty patients (48.4%) underwent total thyroidectomy, whilst twenty-six (41.9%) patients had lobectomy in the NPVCP group and thirty-nine patients (50%) underwent total thyroidectomy, whilst thirty-four (43.6%) patients had lobectomy in the NPVCP group. No significant differences in surgical procedure were found between PVCP and NPVCP groups.

**Table 1:** Characteristics of patients with and without postoperative vocal cord paralysis (PVCP and NPVCP) after thyroid surgery.

Characteristics	NPVCP (n=62)	PVCP (n=78)	P value
Age (years)	46.6±9.1	47.9±10.3	0.643
Gender			
Male	20 (32.3%)	30 (38.5%)	0.482
Female	42 (67.7%)	48 (61.5%)	
Primary diseases			
Papillary thyroid carcinoma	55 (88.7%)	72 (92.3%)	0.562
Nodular goiter	7 (11.3%)	6 (7.7%)	
Surgical procedure			0.775
Total thyroidectomy	30 (48.4%)	39 (50%)	
Lobectomy	26 (41.9%)	34 (43.6%)	
Others	6 (9.7%)	5 (6.4%)	
Preservation of RLN			0.922
Intact	39 (62.9%)	48 (61.5%)	
Sutured after Transected	11 (17.7%)	13 (16.7%)	
Transected	7 (11.3%)	8 (10.3%)	
Unknown	5 (8.1%)	9 (11.5%)	
VHI			
Emotional score	10.13±6.48	15.96±7.26	<0.001
Functional score	16.44±6.73	21.83±8.56	<0.001
Physical score	16.81±7.41	25.37±8.14	<0.001
Total score	41.15±16.32	66.26±22.18	<0.001
Voice acoustic analysis			
Jitter (%)	0.63±0.28	0.86±0.39	<0.001
Shimmer (%)	1.53±0.68	2.19±0.94	<0.001
DSI	-0.43±0.61	-1.32±0.72	<0.001

The data are reported as means±SD or number (percentage). The comparisons of data between the NPVCP and PVCP groups were done by Mann-Whitney test, Fisher's exact test, or chi-square test. RLN: recurrent laryngeal nerve; VHI: Voice Handicap Index; DSI: Dysphonia Severity Index.

**Figure 1:**
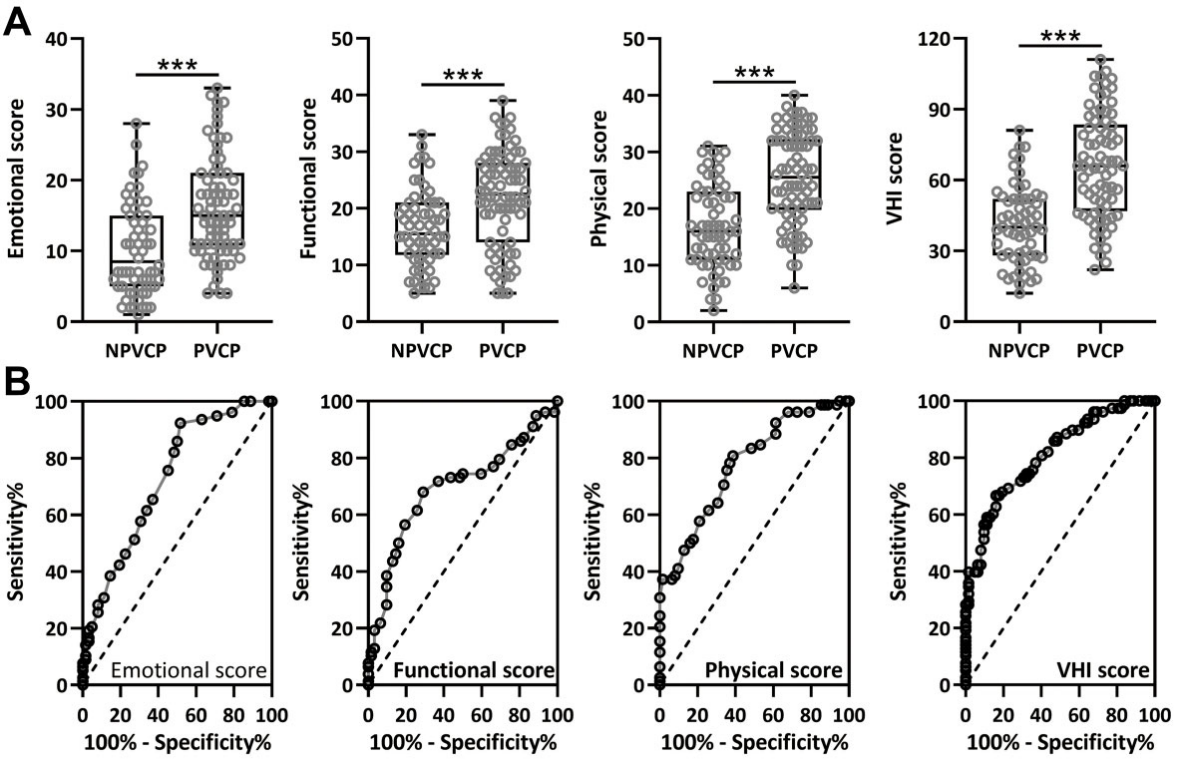
**A**, Voice Handicap Index (VHI) parameters including emotional score, functional score, physical score, and total score between patients with and without postoperative unilateral vocal cord paralysis (PVCP and NPVCP). The data are reported as box plots (median and interquartile range) with all the data points. ***P<0.001 from Mann-Whitney test. **B**, Receiver operating characteristics (ROC) curve analysis showing the diagnostic values of the parameters for PVCP.

In the PVCP group, the VHI including emotional score, functional score, and physical score were significantly higher than in the NPVCP group (Figure 1A).

Moreover, jitter (%) and shimmer (%) parameters were higher in the PVCP than in the NPVCP group (jitter (%): 0.86 *vs* 0.63, P<0.001; shimmer (%): 2.19 *vs* 1.53, P<0.001). In addition, the NPVCP group had a higher DSI than the PVCP group (DSI: -1.32 *vs* -0.43, P<0.001).

### ROC analysis of VHI parameters

ROC analysis revealed that VHI scores were associated with the diagnosis of PVCP (Figure 1B). VHI scores including emotional score, functional score, physical score, and total score yielded AUCs of 0.73, 0.69, 0.78, and 0.81, respectively (Table 2).

**Table 2:** Diagnostic values for postoperative unilateral vocal cord paralysis from receiver operating characteristics curve analysis.

	Cut-off value	AUC (95%CI)	P	Sensitivity (%)	Specificity% (%)	Youden Index
Voice Handicap Index						
Emotional score	7.5	0.73 (0.64 to 0.81)	<0.001	92.31	48.39	0.41
Functional score	19.5	0.69 (0.61 to 0.78)	<0.001	67.95	70.97	0.39
Physical score	17.5	0.78 (0.70 to 0.85)	<0.001	80.77	61.29	0.42
Total score	55.5	0.81 (0.74 to 0.88)	<0.001	66.67	83.87	0.51
Voice acoustic analysis						
Jitter	0.89	0.67 (0.59 to 0.76)	<0.001	48.72	83.87	0.33
Shimmer	2.47	0.71 (0.62 to 0.79)	<0.001	42.31	93.55	0.36
DSI	-0.79	0.84 (0.77 to 0.90)	<0.001	74.36	77.42	0.52

AUC: area under the curve; CI: confidence interval; DSI: Dysphonia Severity Index.

### ROC analysis of voice acoustic parameters

**Figure 2:**
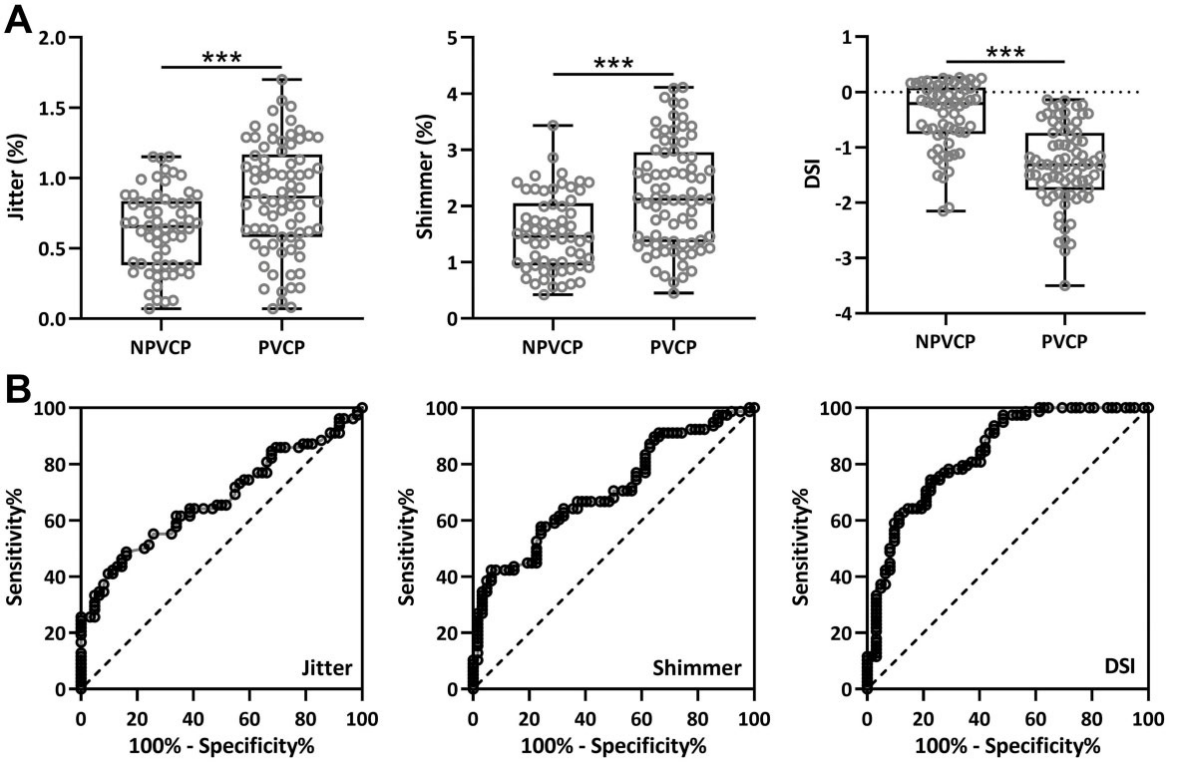
**A**, Voice acoustic analysis compared the parameters including jitter (%), shimmer (%), and Dysphonia Severity Index (DSI) between patients with and without postoperative unilateral vocal cord paralysis (PVCP and NPVCP). The data are shown as box plots with all the data points. ***P<0.001 Mann Whitney test. **B**, Receiver operating characteristics (ROC) curve analysis was performed to determine the diagnostic values of parameters including jitter (%), shimmer (%), and DSI among patients who underwent thyroid surgery.

Furthermore, we compared voice acoustic parameters between PVCP and NPVCP groups, as displayed in Figure 2A. Consistently, jitter (%), shimmer (%), and the DSI score were higher in PVCP than in NPVCP (Figure 2A). Interestingly, ROC analysis revealed that voice acoustic parameters were associated with the diagnosis of PVCP (Figure 2B). An AUC of 0.67, 0.71, and 0.82 were generated by jitter (%), shimmer (%), and DSI, respectively (Table 2). Among them, DSI was highly related to PVCP (AUC=0.84, 95%CI=0.77 to 0.90).

### Correlation analysis of VHI and voice acoustic parameters

**Figure 3:**
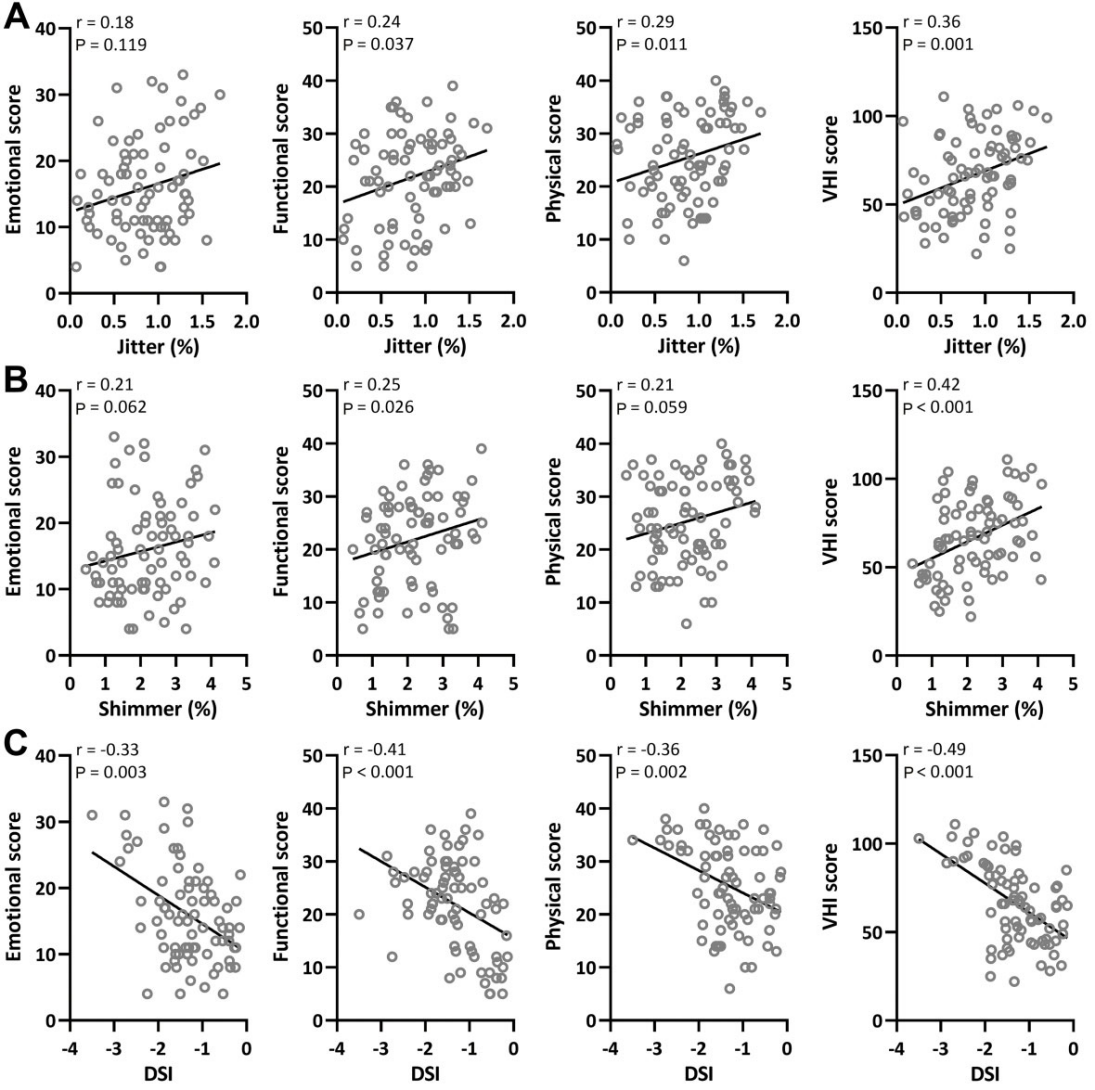
**A**, Spearman correlation analysis displayed the correlation of jitter (%) to Voice Handicap Index (VHI) parameters including emotional score, functional score, physical score, and total score in patients with postoperative unilateral vocal cord paralysis after thyroid surgery. **B**, Spearman correlation analysis displayed the correlation of shimmer (%) to VHI parameters including emotional score, functional score, physical score, and total score in patients with postoperative unilateral vocal cord paralysis after thyroid surgery. **C**, Spearman correlation analysis displayed the correlation of Dysphonia Severity Index (DSI) to VHI parameters including emotional score, functional score, physical score, and VHI total score in patients with postoperative unilateral vocal cord paralysis after thyroid surgery.

We further analyzed the correlation of VHI and voice acoustic parameters, and found a positive correlation between jitter (%) and functional, physical, and VHI scores (Figure 3A). However, the r coefficient ranging between 0.24 and 0.36 suggested a weak correlation between those parameters. Additionally, a positive correlation between shimmer (%) and functional and VHI scores was observed, with VHI score and shimmer (%) having a moderate correlation (Figure 3B). Interestingly, we observed a correlation between DSI and VHI emotional, functional, physical, and VHI total scores (Figure 3C). Among them, DSI had a moderate correlation with VHI score, as suggested by an r value of -0.49.

## Discussion

The VHI has proven to be an effective method to evaluate psychosocial consequences of voice disorders (13,18). Each subscale score ranges between 0 and 40 and the total VHI score ranges between 0 and 120. Previous clinical studies demonstrated the internal consistency and reliability of the VHI in the evaluation of patient perception of voice disorders (19). The results of our study confirmed the reliability of the VHI scores.

In addition, we also evaluated the voice acoustic parameters including jitter (%), shimmer (%), and DSI among patients who underwent thyroid surgery and had vocal cord paralysis. Therefore, we only measured jitter and shimmer and not maximum phonation time, frequency, and intensity. Jitter, shimmer, and DSI results were consistent with the VHI assessment, suggesting that patients in the PVCP group showed worse voice quality.

Subjective and objective assessments are not always consistent in diagnosing voice outcomes (20-22). For instance, in a clinical cohort study of 7 patients with benign vocal cord lesions, no correlation was observed between Praat voice analysis and VHI score (20). Interestingly, in another clinical study of 190 thyroidectomy patients, a significant correlation was observed between GRBAS (grade, roughness, breathiness, asthenia, strain) scores and VHI and acoustic parameters, including shimmer and pitch, after thyroidectomy (23). In this study, we determined the diagnostic values of VHI and acoustic parameters for PVCP. Our results revealed that subjective and objective parameters were associated with the diagnosis of PVCP. Moreover, a positive moderate correlation between VHI score and shimmer (%) was observed. Additionally, DSI had a moderate correlation with functional and VHI scores. These results suggested that a comprehensive assessment (subjective and objective) of voice quality is required for the diagnostic use of PVCP.

Several limitations should be acknowledged in this study. Firstly, our investigation concentrated on patients experiencing vocal cord paralysis following thyroid surgery. Although this provided valuable insights into voice outcomes of this specific subgroup, it restricted the applicability of our findings to a more diverse population undergoing thyroid surgery. Secondly, study duration was limited, emphasizing the need for a more extended follow-up to gain a comprehensive understanding of the sustained impact on voice quality over time. Despite these limitations, our research significantly contributes to the field by demonstrating the diagnostic efficacy of both subjective (VHI) and objective (voice acoustic analysis) measures in the assessment of post-thyroid surgery.

### Conclusions

After thyroid surgery, patients with PVCP showed higher VHI scores and poorer voice acoustic parameters than NPVCP. VHI and total DSI were highly correlated to PVCP. Our findings suggested that VHI scores and acoustic parameters were associated with the diagnosis of PVCP.

## Supplementary Material

Click here to view [pdf].
