# Supplementary material for: Correlation of acoustic voice analysis and Voice Handicap Index in patients with postoperative unilateral vocal cord paralysis after thyroid surgery
Source: Braz J Med Biol Res. 2024 Jun 17;57:e13528. doi: 10.1590/1414-431X2024e13528 (PMC11186591; doi:10.1590/1414-431X2024e13528)
Supplement: Supplementary file 1 [file 1414-431X-bjmbr-57-e13528-suppl.pdf]

Table S1. Voice handicap index (VHI). Participants are provided with statements commonly employed to characterize their voices and articulate the impact of their voices on various aspects of their lives. Please circle the response that indicates how frequently you have the same experience. 0=Never; 1=Almost never; 2=Sometimes; 3=Almost always; 4=Always.

Part I: Functional						
F1	My voice makes it difficult for people to hear me.	0	1	2	3	4
F2	People have difficulty understanding me in a noisy room.	0	1	2	3	4
F3	My family has difficulty hearing me when I call them throughout the house.	0	1	2	3	4
F4	I use the phone less often than I would like to.	0	1	2	3	4
F5	I tend to avoid groups of people because of my voice.	0	1	2	3	4
F6	I speak with friends, neighbors, or relatives less often because of my voice.	0	1	2	3	4
F7	People ask me to repeat myself when speaking face-to-face.	0	1	2	3	4
F8	My voice difficulties restrict personal and social life.	0	1	2	3	4
F9	I feel left out of conversations because of my voice.	0	1	2	3	4
F10	My voice problem causes me to lose income.	0	1	2	3	4
Part II: Physical						
F1	I run out of air when I talk.	0	1	2	3	4
F2	The sound of my voice varies throughout the day.	0	1	2	3	4
F3	People ask, "What's wrong with your voice?"	0	1	2	3	4
F4	My voice sounds creaky and dry.	0	1	2	3	4
F5	I feel as though I have to strain to produce voice.	0	1	2	3	4
F6	The clarity of my voice is unpredictable.	0	1	2	3	4
F7	I try to change my voice to sound different.	0	1	2	3	4
F8	I use a great deal of effort to speak.	0	1	2	3	4
F9	My voice is worse in the evening.	0	1	2	3	4
F10	My voice "gives out" on me in the middle of speaking.	0	1	2	3	4
Part III: Emotional						
F1	I am tense when talking to others because of my voice.	0	1	2	3	4
F2	People seem irritated with my voice.	0	1	2	3	4
F3	I find other people don't understand my voice problem.	0	1	2	3	4
F4	My voice problem upsets me.	0	1	2	3	4
F5	I am less outgoing because of my voice problem.	0	1	2	3	4
F6	My voice makes me feel handicapped.	0	1	2	3	4
F7	I feel annoyed when people ask me to repeat.	0	1	2	3	4
F8	I feel embarrassed when people ask me to repeat.	0	1	2	3	4
F9	My voice makes me feel incompetent.	0	1	2	3	4
F10	I am ashamed of my voice problem.	0	1	2	3	4
